# The Barwon Health Eating Disorder Service

**DOI:** 10.1186/2050-2974-1-S1-O1

**Published:** 2013-11-14

**Authors:** Hollie Laver

**Affiliations:** 1 Barwon Health Service, Australia

## 

The Barwon Health Eating Disorder Service is a specialist regional service providing assessment, treatment and consultation for individuals with eating disorders of all ages living in the Barwon region. The service delivers evidence-based treatments including Family-Based treatment, Cognitive Behaviour Therapy-Enhanced and Cognitive Analytic Therapy in conjunction with dietetic support. The service prides itself on engagement and partnerships with the service providers in the region both in the public and private sector to provide the community with a range of integrated options for treatment and support.

In recent times the service has collaborated with The Barwon Health Deakin Psychology Clinic and the Community Adult Mental Health teams to provide an assessment clinic and evidence-based outpatient treatment to individuals 26 years and over and their families with eating disorders. This presentation will showcase the work being done in the service and the ongoing development of the adult treatment model in conjunction with service partners, including the important partnership with the Victorian Centre of Excellence in Eating Disorders.

This abstract was presented in the **Adult Treatment and Services** stream of the 2013 ANZAED Conference.

